# METALS (Cr, Mn, Co, Ni) CONCENTRATION IN THE BLOOD PLASMA AND URINE OF POLISH WELDERS AND TELOMERE LENGTH AS AN POTENTIAL INDICATOR OF TOXICITY OF METALS WELDING FUMES EXPOSURE

**DOI:** 10.13075/ijomeh.1896.02493

**Published:** 2025

**Authors:** Wojciech Wąsowicz, Beata Janasik, Edyta Reszka, Edyta Kasperczyk, Jędrzej Chrzanowski, Wojciech Fendler

**Affiliations:** 1 Nofer Institute of Occupational Medicine, Department of Biological and Environmental Monitoring, Łódź, Poland; 2 Nofer Institute of Occupational Medicine, Central Laboratory, Łódź, Poland; 3 Nofer Institute of Occupational Medicine, Department of Chemical Safety, Łódź, Poland; 4 Nofer Institute of Occupational Medicine, Department of Translational Research, Łódź, Poland; 5 University of Lodz, Department of Biophysics of Environmental Pollution, Faculty of Biology and Environmental Protection, Łódź, Poland; 6 Medical University of Lodz, Department of Biostatistics and Translational Medicine, Łódź, Poland

**Keywords:** biological monitoring, metals, welders, urine, welding fumes, telomere length

## Abstract

**Objectives:**

The study investigated the concentrations of metals (chromium [Cr], manganese [Mn], cobalt [Co], nickel [Ni]) in the blood plasma and urine of Polish welders exposed to these elements contained in welding dust/fumes based on the results of biological monitoring, analyze the interrelationships between these elements, and attempt to correlate these data with telomere length. It is believed that telomere length can be considered a marker of exposure, including occupational. Analysis of questionnaire surveys was also taken into consideration.

**Material and Methods:**

The study included 118 male welders and 51 age-matched male controls. Metals analysis in plasma and urine were determined by ICP-MS technique. Telomere length was measured in blood genomic DNA using the qRT-PCR method.

**Results:**

Welders had significantly higher plasma levels of Cr, Ni, and Mn (p < 0.0001, respectively). Total concentrations of Cr, Ni, and Mn in the urine of pre-shift subjects were significantly higher compared to controls. Cobalt concentration in urine of exposed welders was significantly higher (p < 0.02) than in control group. Telomere length was exactly the same in the welder group compared to the control (mean ± standard deviation 0.99±0.41 vs. 0.99±0.52, respectively). Plasma and urine metal concentrations and telomere length were also studied in groups of welders in relation to personal protection equipment. Differences were found in plasma and urine metal concentrations according to the aspirators used. Statistically significant linear correlations were found between plasma and urine concentrations of the determined elements both before and after the work shift.

**Conclusions:**

The findings suggest a positive relationship between Ni and Mn (endshift) concentrations and telomere length, the effect which remained statistically significant even after adjusting for age and metabolic status. This indicates a complex interplay between metal exposure and biological aging markers. However, the relationship between exposure to welding fumes and changes in telomere length in welders requires further in-depth research.

## Highlights

- Metals in urine are higher in preshift compared to levels before the work shift.
- Urine metals are dependent on the personal protective equipment used.
- Telomere length is not a marker of exposure to welding fumes.

## INTRODUCTION

An estimated 110 million workers are exposed to welding fumes worldwide. Welding fumes is one of the most important and dangerous hazards to a welder's health. In March 2017, International Agency for Research on Cancer (IARC) convened a Working Group to systematically review all of the published literature to date and classified welding fumes as carcinogenic to humans (group 1), based on sufficient evidence of lung cancer from epidemiological studies [1–3]. Welding dusts are characterized by a very diverse chemical composition and morphology, which determines their toxicity [4]. The content of neurotoxic manganese (Mn), carcinogenic of hexava-lent chromium (Cr(VI)) and nickel (Ni) and other toxic compounds causes that exposure to welding dust is one of the important factors of the increased of the risk of respiratory diseases, including cancers, but concentrations of other metals (Mn, Co) are toxic as well [5]. Currently used biomarkers, both exposures (e.g., blood and urine concentrations), as well as early effects (e.g., oxidative stress markers) are not sufficiently helpful in assessment of toxic/harmful effects of welding fumes metals.

Welders face a number of real and dangerous risks that could significantly impact their current and future quality of life. Welding fumes can cause serious health problems for workers exposed by inhalation to their components [6]. Short-term exposure can result in nausea, dizziness, or eye, nose and throat irritation. Chronically exposure to welding fumes can lead to cancer of the lung, larynx and urinary tract, as well as nervous system and kidney damage. Welding dust is a mixture of fumes of inorganic compounds (metals) and gas by-products [7]. They are formed when a metal is heated above its boiling point and its vapours condense into fine particles. These particles then stay suspended in the vapour of gas. The most common metals in the welding dust are aluminium, arsenic (As), copper, lead (Pb), Mn, zinc, Cr, Ni, depending on the welding methods used and the alloys used. The health consequences for welders depend mainly on the composition of welding fumes.

Welders are exposed to a complex of chemical mixtures that can vary depending on the type of welding method used (e.g., gas, arc), the type of welded metal (mild steel or stainless steel) and the working conditions in which welding is performed. For example, Ni compounds and Cr, well-established lung carcinogens in humans, are constituents of stainless steel whereas they exist in much lower concentrations in mild-steel.

Manganese is a transition metal, occurring at various degrees of oxidation, with diametrically different functions and activities. On the one hand, it is a component and activator of important enzymes, it participates in the bio-synthesis of amino acids, cholesterol and carbohydrate metabolism, on the other hand it has a highly toxic elements. Potential exposure to Mn occurs whenever Mn is used in electrode cores and coatings or in electrode wire. Exposure to fume from welding on Mn steel may give rise acute pneumonia. Chronic exposure to Mn is the cause of nervous system disorders, leads to central nervous system dysfunction, which is preceded by psychological, emotional and psychomotor changes. These changes lead to the development of a chronic neurological disorder called “manganism” [8].

Cobalt (Co) and its compounds are widely distributed in nature. Although Co has a biologically necessary role as a component of vitamin B_12_, excessive exposure has been shown to cause adverse health effects. Because of its widespread occurrence, humans are frequently exposed to various Co compounds in daily life.

The general population is exposed primarily by inhaling ambient air and ingesting food and drinking water containing compounds of Co. Occupational exposure to Co is a relatively frequent occurrence because Co has many industrial applications (hard metal production, grinding, extraction). Cobalt is the component of high-strength steel alloys, hence the inhalation of Co dust is an important risk factor during welding operations stainless steel. Exposure may cause shortness of breath, cough and pneumonia. In contrast to Mn, Cr and Ni, hypersensitivity to Co appears to be reversible, as changes in the lungs occur at low frequency and vary in intensity and timing. In most cases, the symptoms disappear after exposure [9].

Nickel has no significant physiological significance for higher organisms. It is the 24th element in the frequency of occurrence in the earth's crust, so people are constantly exposed to this ubiquitous element [10]. Nickel compounds are known to be a human carcinogens. Metallic Ni are listed as “reasonably anticipated to be a human carcinogen.” Both forms are found in the welding fumes of Ni-plated mild steel and stainless steel electrodes and high-strength low-alloy steel [11]. One of the toxic mechanisms of action of Ni is the generation of free radicals (reactive oxygen species − ROS), oxidative stress reactions and consequent damage to DNA, proteins, lipids, which are the beginning of the process of carcinogenesis [12]. *In vitro* studies confirm the toxic effects of Ni, included especially form of nano [13,14].

Chromium in the human body plays an important role, it is an essential element. It can occur at various oxidation levels: 0, II, III, IV, V and VI, but form III and VI being the most important for humans. Chromium(III) is a factor in the normal metabolism of glucose, it is part of the glucose tolerance factor. It plays an important role in the metabolism of some proteins and lipids, especially cholesterol [15]. The occurrence of Cr(VI) is rare naturally. Most of Cr(VI) compounds are man-made (products or by-products) and human-caused Cr(VI) contamination is the result of large industrial emissions (mainly from metallurgy, chemicals, and refractory brick industries) and dyes production, corrosion inhibitors, chemical synthesis, production of refractories, leather tanning, and wood preservation [16]. Breathing contaminated air in the workplace is the main source of exposure in occupational settings. For the general population, exposure to Cr occurs mainly as a result of consumption of contaminated soil, food and water, but also by through inhalation of ambient air. Smoking is another important source of exposure to hexavalent Cr.

However, in addition to the general population, in EU the estimated number of Cr(VI)-exposed workers in 2012 was about 786 000, with the largest numbers exposed to welding [3]. In the EU CLP Regulation (EC) No. 1272/2008 they are classified as genotoxic and as carcinogen. Chromium(VI) is present in the welding fumes arising from the treatment of stainless steel or in the fumes from weld electrodes and Cr alloys. It has been reported that exposure to vapours containing high concentrations of water-soluble Cr(VI) when welding stainless steel in confined spaces causes both acute and chronic Cr poisoning, dermatitis and asthma. Epidemiological studies and animal tests have confirmed that certain Cr(VI) compounds are occupational carcinogens [17]. The assessment of exposure to toxic metals found in welding fumes/dust, in addition to the obligatory monitoring of the work environment, is also based on the use of biological monitoring tools. The basic assessment uses biomarkers of exposure, i.e., analysis of metals in biological material. However, biomarkers of effects can be an equally important tool as predictors of exposure to toxic agents occurring during welding processes.

Currently, among the new molecular markers used in bio logical monitoring, medicine and diagnostics, among others, the authors can distinguish the study of telo-mere length (TL) as a marker that can indicate the lifespan of cells. Telomere length can be an indicator of the aging of the body, the development of certain diseases including cancer [18]. Telomeres are complexes of tandem repeats of DNA (TTAGGG) and protein that cap eukaryotic chromosomes and play a critical role in chromosome stability [19]. Telomere length is determined, among other things, by mechanisms occurring during the replication process and varies from 2 to 10 kilobase pairs (kbp) [20]. Telomere length may be affected by environmental and occupational exposure to chemicals. Lifestyle factors and occupational stressor can accelerate telo-mere shortening or induce increase in TL [21]. Oxidative stress and inflammation are important pathways for such diseases, and are also risk factor for TL shortening [19]. Most studies have shown shorter telomeres in subject exposed to polycyclic aromatic carbons, pesticides or cadmium (Cd). Some toxic agents such as As or persistent organic pollutants (POPs) have been associated with longer telomeres [22]. Nonetheless, a general agreement has been established that measuring TL offers valuable insights and forms a crucial foundation for analyzing gene expression and epigenetic data, although published data are inconclusive. Exposure to welding fumes can cause free radical activity in tissues [23,24]. In the body, free radicals can cause DNA damage which is suggested as a possible mechanism of cancer development caused by exposure to welding fumes [25,26]. It has been hypothesised that oxidative stress may be the basic mechanism responsible for changes observed in TL [27,28], and may be used as bio-marker of cancer risk. There are few articles treating the study of telomere length in occupational exposure to welding dust, tobacco smoke, or pesticides.

To date, no such studies have been carried out involving workers exposed to welding fumes in Poland, and published results from other centres around the world are scarce, and sometimes controversial [29,30].

Therefore, the purpose of this study was to determine the concentrations of metals (Cr, Mn, Co, Ni) in the blood plasma and urine of Polish welders exposed to these elements contained in welding dust/fumes based on the results of biological monitoring, analyse the interrelationships between these elements, and attempt to correlate these data with TL. The welding technique will also be taken into consideration, as well as the worker protection devices used. Analysis of questionnaire surveys was also taken into consideration.

## MATERIAL AND METHODS

The cohort included 118 male welders, who were employed at 8 plants, experienced occupational exposure to Cr, Mn, Co, and Ni at workplace, and 51 age-adjusted males not exposed to these elements, as a controls. Research was conducted in 2018–2022. Environmental exposure to these metals in welders and controls were similar. Approval was obtained from the Ethics Committee operating at the Nofer Institute of Occupational Institute, Łódź, Poland to conduct the study (No. 07/2018). In addition, written consent was obtained from all subjects (welders and controls) to participate in the study, and after being warned that they could withdraw from the study at any time.

The characteristics of exposed and controls group are shown in Table 1.

**Table 1.** Characteristics of the population exposed to welding fumes and the control population in welding companies in Poland, 2018–2022

Variable	Participants (N = 169)				p
	exposed (N = 118)		controls (N = 51)		
	M±SD	n (%)	M±SD	n (%)	
Age [years] (exposed N = 115^*^, controls N = 51)	42.52±9.93		43.20±11.97		0.7059
Height [cm]	177.47±6.58		177.71±7.29		0.8301
Body weight [kg]	88.72±12.41		84.85±11.25		0.0610
BMI [kg/m^2^]	28.14±3.35		26.90±3.51		0.0337
Cigarette smoking					0.6808
yes		78 (66.1)		34 (69.4)	
no		40 (33.9)		15 (30.6)	
Alcohol drinking (exposed N = 53, controls N = 13)					0.7643
yes		48 (90.6)		12 (92.3)	
no		5 (9.4)		1 (7.7)	
Duration of exposure [years] (exposed N = 108^*^, controls N = 0)	16.63±10.67		–		–

* Differences in data are due to non-response in the questionnaires.

### Collection of contextual information

General information on the workplace, work practices and risk management measures (RMMs) were collected from a company representative prior to the sampling campaign. Detailed interviews were conducted by interviewers to obtain information of employment, smoking, alcohol consumption, work task, type of welding metal inert gas (MIG), tungsten inert gas (TIG) and other (the other group included 32 welders who welded with different methods during the working day and so the welding method could not be strictly defined), personal protection equipment (PPE), occupational histories and other exposures at work. The concentrations of metals in the urine and plasma of welders were also studied in relation to the personal protective equipment used (helmets with different types of absorbers). Samples were taken before and after the work shift.

### Blood and urine sampling

#### Blood sampling

Venous blood samples (7 ml) were collected from workers into test tubes, trace elements free, to avoid the background contamination. Blood samples was collected, preferentially on the third to fifth day of the working week, at the end of the work shift. Blood samples was collected used to a tube appropriate for trace element analyses containing potassium ethylenediaminetetraacetic acid (K-EDTA) as anticoagulant. Separation of plasma was conducted, preferably within 8 h (and max. 24 h) from the specimen collection. Samples were centrifuged (10 min at 1000–2000 × g or 5 min at 2700 × g) and the supernatants containing the plasma were stored at −20°C until analysis.

#### Urine sampling

Two spot urine samples (20 ml each) were collected from the exposed workers, the first before the start of the shift, and the second one at the end of the shift in the end of the working week. Urine samples were collected in previously decontaminated containers (e.g., pre-washed with 10% of nitric acid solution) to avoid background contamination. After collection, urine samples were homogenized and aliquoted in several prelabeled tubes and stored at −20°C. Urinary creatinine (Ct) concentrations were measured and metal levels were normalized to Ct.

#### Blood and urine samples form controls

One blood and urine sample was collected from each non-exposed subjects, at any time of the working week.

#### Creatinine determination

According to the WHO the acceptable Ct concentration range of the urine specimen is 0.3–3.0 g/l [31]. Creatinine levels were determined in group of welders and controls using colorimetric Jaffe method [32]. Analysis was carried out at 520 nm on Lambda EZ210 spectrometer (Perkin-Elmer, Waltham, MA, USA).

#### Air sampling

Air samples were collected as previously described by Stanislawska et al. [33].

#### Metals determination in air

Metals determination was prepared as previously described by Stanislawska et al. [33,34]. The laboratory participates in the Interlaboratory Quality Control Programme for Metals on Filters (PICC-MET) organised by the National Centre for Work Conditions in Barcelona, Spain.

#### Metals determination in biological material

Levels of Cr, Ni, Co, and Mn in plasma and urine were determined by an inductively coupled plasma mass spectrometry (ICP-MS, NEXion 350D, PerkinElmer). Clin-Check^®^ Urine Control for Trace Elements (RecipeChemicals + InstrumentsGmbH, München, Germany) and ClinCheck^®^ Serum Control for Trace Elements (Recipe Chemicals + Instruments GmbH) were used for internal quality control for urine and plasma respectively. Internal quality control were analysed during each run. Chromium, Ni, Co, and Mn levels in urine were normalized to Ct. The laboratory Institute participates in the German External Quality Assessment Scheme (G-EQUAS) for metals determination organized by the Institute and Out-Patient Clinic for Occupational, Social and Environmental Medicine of the Friedrich-Alexander University of Erlangen-Nuremberg in Erlangen, Germany.

#### Analysis of telomere length

First, genomic DNA was isolated. It was extracted form whole blood of 51 welders and 51 controls using the QIAamp DNA Blood Mini Kit (QIAGEN, Hilden, Germany) according to the manufacture protocol. Concentration and purity of DNA was checked using a NanoDrop photometer (ThermoFisher Scientific, Waltham, MA, USA).

Then, TL and the reference gene *FTH1*, coding for ferritin heavy chain 1 in samples of genomic DNA were determined. Telomere length was measured in blood genomic DNA using the qRT-PCR method with SYBRGreen (Roche, Basel, Swizerland) dye as described by O’Callaghan et al. [35] with modifications of Erdem et al. [36]. The method uses measurement of absolute TL based on the telomere length standard curve. Telomere length was determined using standard curves for TL copy number and *FTH1* using telomere length standard − pUC57 DNA plasmid with insert for TL and *FTH1*.

The primer sequences were as follows: TL forward (F): CGGTTTGTTTGGGTTTGGGTTTGGGTTTTTGGGTTTGGG TT-3’, TL reverse (R): 5’-GGCTTGCCTTACCCTTACCCTTACCCTTACCCTTACCCT-3’, *FTH1* F: 5’-GATGATGTGGCTTTGAAGAACTTTGCCA-3’, *FTH1* R: 5’-CACCTCGTTGGCTGCAGCTTCATCA-3’.

The specificity of the primer was determined by melting temperature analysis. Quantitative PCR (qPCR) was performed using DNA template in a total volume of 10 μl. Telomere length, characterized by a single copy, and the reference gene *FTH1* with multiple copies present in the genome were amplified using a FastStart SYBR Green Master (Roche) and an LC96 real-time thermocycler (Roche). A standard curve was generated by performing serial dilutions of plasmid DNA containing a 600 bp telomere sequence and 1 copy of the *FTH1* sequence. Plasmid DNA pUC57 (GenScript, Piscataway, NJ, USA) was added to each standard to maintain a constant amount of total DNA per reaction. A standard dilution was prepared to ensure minimal variability between different runs. The adjusted coefficient of determination (r^2^) values for the standard curves for TL and *FTH1* for experiments were >0.994. Telomere length value in kbp from each sample was calculated from standard curve and expressed as total telomeric length per genome (kbp/genome) and additionally converted to relative units.

### Statistics

Categorical variables were reported as percentages and numbers and compared using the χ^2^ test or Fisher's exact test with or without Yates continuity correction, as appropriate. Continuous variables were checked for normality using the Shapiro-Wilk test. For normally distributed variables, means (M) and standard deviations (SD) were reported, and for non-normally distributed, medians (Me) and interquartile ranges (IQR) were used. Continuous variables were compared between groups using either the Student's t-tests and ANOVA with Tukey's *post hoc* (for >2 groups) or the Mann-Whitney U test and Kruskall-Wallis test with Dunn's *post hoc* (for >2 groups), depending on satisfying the normality assumption. For the paired comparisons (pre-end shift), a paired t-test or Wilcoxon test was used, depending on satisfying the normality assumption. Pearson's correlation coefficients were calculated to assess the relationships between continuous variables, including correlation with change in paired comparisons.

For the association of telomer length with the metal concentrations and clinical variables, univariate and multivariate (to adjust for clinical covariates) regression was conducted for each variable. Then, the final multivariate linear regression model was determined as the best subset of metal concentrations and clinical variables.

Statistical significance was defined as a p-value <0.05. All statistical analyses were performed using Statistica 13.3 (TIBCO Software, Palo Alto, CA, USA) and Python 3.11 with statsmodels and scikit-learn packages. Graphical representations of the data were created using Statistica 13.3 (TIBCO Software) and Python 3.11 with Matplotlib and Seaborn packages.

## RESULTS

The study included 118 male welders and 51 age-matched male controls.

The demographic and clinical characteristics of the study participants are summarized in Table 1. The age of the welders was M±SD 42.5±9.9 years, while the age of the controls was M±SD 43.2±12.0 years (p = 0.71). Welders had a significantly higher BMI compared to controls (M±SD 28.1±3.4 kg/m² vs. 26.9±3.5 kg/m², p = 0.03). The prevalence of smoking and alcohol consumption was similar between the 2 groups.

### Industrial hygiene samples (air)

In light of the applicable legal regulations in Poland [37], comparing the results of the metal concentrations in the work environment the airborn Mn did not exceeded the maximum acceptable concentration (MAC) value (0.05 mg/m^3^). The MAC of Cr(VI) (0.01 mg/m^3^) was exceeded in 2 cases. The concentrations of Ni was exceeded in 4 cases.

### Biological material

The concentrations of metals (Cr, Mn and Ni) in blood plasma and Cr, Ni Mn and Co in urine and TL in blood are presented in Table 2. Welders had significantly higher plasma levels of Cr, Ni, and Mn than those observed in unexposed group (p < 0.0001). Total concentrations of Cr, Ni, and Mn in the urine of pre-shift subjects both expressed in μg/l and in μg/g of Ct were significantly higher compared to the values observed in the unexposed group. Cobalt concentration in urine of exposed welders was significantly higher as expressed in μg/l (p < 0.02), and slightly higher, but not significant after Ct adjustment, as compared to controls (M±SD 0.28±0.27 μg/g Ct vs. 0.25±0.15 μg/l, n.s.). Creatinine content was significantly higher in welders as compared to controls (p < 0.05). Total concentrations of Cr, Ni, and Mn in the urine of pre-shift subjects both expressed in μg/l and in μg/g of Ct were significantly higher compared to the values observed in the unexposed group. Cobalt concentration in urine of exposed welders was significantly higher as expressed in μg/l (p < 0.02), and slightly higher, but not significant after Ct adjustment (M±SD 0.28±0.27 μg/g Ct vs. 0.25±0.15 μg/l, n.s.). Telomere length was exactly the same in the welder group compared to the control (M±SD 0.99±0.41 vs. 0.99±0.52, respectively) (Table 2).

**Table 2.** Chromium (Cr), nickel (Ni) and manganese (Mn) concentration in plasma and urine and telomere length (TL) in exposed workers and controls in welding companies, Poland, 2018–2022

Parameter	Participants (N = 169)			p
	exposed (N = 118)		controls (N = 51)	
	M±SD	n^a^	M±SD	
In plasma [μg/I]				
Cr	0.60±0.26	118	0.46±0.31	0.0001
Ni	1.89±1.52	118	0.84±0.27	0.0001
Mn	1.34±1.04	101	0.82±0.22	0.0001
In urine				
total Cr (pre-shift)				
μg/I	1.87±2.07	113	0.32±0.17	0.0001
μg/g Ct	1.39±1.70	113	0.21±0.20	0.0001
Ni (pre-shift)				
μg/I	3.44±2.66	105	1.32±0.79	0.0001
μg/g Ct	2.19±1.82	112	1.25±0.88	0.0005
Mn (pre-shift)				
μg/I	0.59±0.43	107	0.36±0.23	0.0057
μg/g Ct	0.45±0.45	114	0.29±0.19	0.002
Co (pre-shift)				
μg/I	0.45±0.46	106	0.32±0.20	0.02
μg/g Ct	0.28±0.27	115	0.25±0.15	n.s.
creatinine (pre-shift) [g/I]	1.52±0.68	117	1.32±0.44	0.05
TL^b^				
absolute (aTL) [kb/genome]	91.08±40.48	51	91.08±47.84	n.s.
relative (rTL)	0.99±0.41	51	0.99±0.52	n.s.

Ct-creatinine.

n.s. – not significant.

a Differences in data are the result of statistical analysis.

b O'Callaghan et al. method [35],

Paired comparisons of metal concentrations in urine preand end-shift samples showed significant increases for total Cr (after Ct adjustment, p < 0.04), Ni (after Ct adjustment, p < 0.0009), Mn (without Ct adjustment, p < 0.02) and Co (after Ct adjustment, p < 0.01). The urinary Ct concentration of the subjects before the start of the work shift was 12% higher than after the shift. This difference was statistically significant (p < 0.004) (Table 3).

**Table 3.** Concentration of chromium (Cr), nickel (Ni), manganese (Mn), cobalt (Co) and creatinine (Ct) in urine of welders before and after the work shift in welding companies, Poland, 2018–2022

Parameter	Participants (N = 118)				p
	pre-shift		end-shift		
	M±SD	n^a^	M±SD	n^a^	
Total Cr					
μg/I	1.86±2.06	113	2.16±2.83	113	0.0636
μg/g Ct	1.39±1.70	113	2.03±4.49	113	**0.0420**
Ni					
μg/I	3.44±2.66	105	3.81+3.01	105	0.2014
μg/g ct	2.19±1.81	112	3.26±3.63	112	**0.0009**
Mn					
μg/I	0.53±0.33	107	0.64±0.47	107	**0.0205**
μg/g Ct	0.45±0.45	114	0.58±0.57	114	0.0551
Co					
μg/I	0.44.4776	106	0.48±0.41	106	0.3949
μg/g Ct	0.28±0.27	115	0.39±0.48	115	**0.0136**
Creatinine [g/I]	1.52±0.67	118	1.34±0.55	117	**0.0037**

Bolded are significant values.

a Differences in data are due to statistical analysis.

### Determinants of exposure

Welders using different welding methods (MIG, TIG, and other) exhibited varying metal concentrations in urine. The most common welding processes reported were: MIG (56.8%), TIG (24.6%) and other (11.0%). The highest concentrations of the tested metals were observed in a group of TIG welding workers. Notably, significantly higher concentrations were found in urine total Cr (pre-shift, even after Ct adjustment), urine Ni (pre-shift, even after Ct adjustment), total Cr (end-shift expressed as μg/l), and Mn (end-shift after Ct adjustment). *Post hoc* analysis showed further statistically significant differences in metal concentrations depending on the welding method (Table 4). The concentration of Mn in the plasma differed significantly between the MIG, TIG and other groups, while differences in the concentrations of the other metals showed no statistically significant differences). No statistical differences was found in TL between tested group (Table 4).

**Table 4.** Chromium (Cr), nickel (Ni), and manganese (Mn) concentration in plasma and Cr, Ni, Mn, and cobalt (Co) in urine and telomere length (TL) of welders depending on the welding techniques and the use of personal protective equipment (PPE) in welding companies, Poland, 2018–2022

Parameter	Welding technique					PPE		
	MIG (N = 67)	TIG (N = 16)	other (N = 32)	p	*post hoc^a^*	with filter (N = 29^b^)	without filter (N = 78^b^)	p
In plasma [μg/I] (M±SD)								
Cr	0.45±0.33	–	0.49±0.29	0.638	–	0.38±0.20	0.77±0.44	**0.0005**
Ni	1.51±0.88	2.16±1.87	1.61±0.47	0.106	n.s.	1.33±0.46	2.08±1.75	**0.02**
Mn	0.88±0.31	1.82±1.29	0.82±0.18	**<0.0001**	1, 2; 1, 3	0.76±0.19	1.66±1.22	**0.0002**
In urine (M±SD)								
total Cr								
pre-shift								
μg/I	0.93±0.88	2.47±2.42	1.55±1.63	**0.002**	1, 2	0.84±0.53	2.48±2.32	**0.0003**
μg/g Ct	0.74±0.69	1.76±1.98	1.36±1.78	**0.022**	1, 2	0.73±0.65	1.81±1.95	**0.0047**
end-shift								
μg/I	0.95±0.85	3.04±3.51	1.26±0.77	**0.001**	1, 2; 1, 3	0.93±0.63	2.86±3.28	**0.0023**
μg/g Ct	0.93±0.86	2.86±5.89	1.23±1.17	**0.108**	1, 2	0.89±0.72	2.71±5.45	0.0771
Ni								
pre-shift								
μg/I	2.38±1.20	3.98±3.16	2.89±0.90	**0.024**	1, 2	2.58±1.08	3.93±3.00	**0.0329**
μg/g Ct	1.90±1.52	2.56±2.02	1.31±1.25	**0.030**	1, 3	2.08±1.51	2.36±2.00	0.4927
end-shift								
μg/I	3.21±2.42	4.06±3.21	4.60±3.10	0.257	n.s.	4.02±3.22	3.94±3.03	0.8968
μg/g Ct	3.00±2.18	3.47±4.44	3.53±2.25	0.816	n.s.	3.56±2.61	3.38±4.13	0.8335
Mn								
pre-shift								
μg/I	0.45±0.17	0.72±0.52	0.42±0.12	**0.003**	1, 2; 1, 3	0.42±0.16	0.68±0.49	**0.0077**
μg/g Ct	0.38±0.23	0.53±0.57	0.32±0.16	0.166	n.s.	0.38±0.24	0.50±0.53	0.2262
end-shift								
μg/I	0.48±0.30	0.90±0.65	0.41±0.11	**0.001**	1, 2; 1, 3	0.50±0.30	0.82±0.62	**0.0126**
μg/g Ct	0.50±0.47	0.69±0.66	0.32±0.17	**0.045**	1, 3	0.52±0.49	0.64±0.62	0.3644
Co								
pre-shift								
μg/I	0.36±0.29	0.49±0.57	0.49±0.29	0.823	n.s.	0.44±0.34	0.48±0.53	0.7224
μg/g Ct	0.26±0.19	0.29±0.32	0.31±0.17	<0.0001	n.s.	0.31±0.20	0.29±0.30	0.7852
end-shift								
μg/I	0.23±0.15	0.70±0.51	0.40±0.28	<0.0001	1, 2, 3	0.33±0.26	0.62±0.50	**0.0046**
μg/g Ct	0.29±0.56	0.48±0.494	0.26±0.15	0.099	n.s.	0.35±0.59	0.44±0.46	0.4123
Ct [g/I]								
pre-shift	1.33±0.57	1.61±0.75	1.52±0.49	0.156	n.s.	1.31±0.58	1.60±0.73	0.0618
end-shift	1.15+0.50	1.40±0.53	1.39±0.65	**0.102**	1, 2	1.19±0.58	1.37±0.53	0.1491
TL^c^								
absolute (aTL) [kb/genome]	98.44±40.48	–	81.88±30.36	0.176	n.s.	100.28±37.72	84.64±44.16	n.s.
relative (rTL)	1.07±0.44	–	0.89±0.33	0.176	n.s.	1.09±0.41	0.92±0.48	n.s.

Ct – creatinine.

MIG – metal inert gas; TIG – tungsten inert gas.

n.s. – not significant.

Bolded are significant values.

a Significance for *post hoc* tests was marked with 2 digit number combinations for specific *post hoc*, with 1 corresponding to MIG, 2 toTIG, and 3 to other.

b For TL the number was 51.

c O'Callaghan et al. method [35],

The use of helmets with filter (PPE) was associated with significantly lower concentrations of Cr, Ni, Mn in plasma (M±SD 0.38±0.20 μg/l vs. 0.77±0.44 μg/l, p < 0.0005 for Cr; 1.33±0.46 μg/l vs. 2.08±1.75 μg/l, p < 0.02 for Ni; 0.76±0.19 μg/l vs. 1.66±1.22 μg/l, p < 0.0002 for Mn) as compared with the data obtained from welders those working without PPEs. It was found total Cr (pre-shift even after Ct adjustment, end shift without adjustment), Ni (pre-shift without adjustment), Mn (pre-shift and end-shift, both without adjustment), Co (end-shift without adjustment)concentrations in urine of welders using helmets with filters were significantly lower compared to data obtained in the urine of those not using PPEs (Table 4).

Concentrations of metals in urine and plasma of welders were also studied depending on the PPE used (helmets with different types of absorbers). Statistically significant differences were found in the concentrations of Cr, Ni, Mn and Co in the urine of those exposed depending on the PPEs used. The highest concentrations of the metals tested were observed in the urine of welders using a helmet without any respirator. A decrease in the concentrations of the determined metals was observed depending on the respirators used. The lowest concentrations of the labeled parameters were found in the urine of welders using a welding helmet with powered or air fed, filtering respirator (Table 5).

**Table 5.** Chromium (Cr), nickel (Ni), manganese (Mn) and cobalt (Co) concentration in plasma and urine of welders who did and did not utilize personal protective equipment (PPE), Poland, 2018–2022

Parameter	PPE – welding helmet					
	without any respirator (N = 78)	with half mask re-usable dust respirator (N = 11)		with disposable particulate respirator (N = 3)	with powered or air fed, filtering respirator (N = 15)	
	M±SD	M±SD	p	M±SD	M±SD	p
In plasma [μg/I]						
Cr	0.78±0.44	0.54±0.26	<0.02^a^	0.31±0.06	0.29±0.09	<0.001^b^, <0.01^c^
Ni	2.09±1.76	1.20±0.52	<0.001^a^	1.68±0.29	1.34±0.44	<0.001^b^, n.s.^c^
Mn	1.77±1.27	0.82±0.24	<0.001^a^	0.76±0.07	0.73±0.17	<0.001^b^, n.s.^c^
In urine						
total Cr						
pre-shift						
μg/I	2.48±2.33	1.26±0.48	<0.002^e^	0.92±0.24	0.53±0.38	<0.001^d^, <0.001^f^
μg/g Ct	1.81±1.97	1.21±0.87	n.s.	0.48±0.06	0.44±0.20	<0.001^d^, <0.001^f^
end-shift						
μg/I	2.86±3.31	1.40±0.62	<0.001^e^	0.91±0.55	0.60±0.44	<0.001^d^, <0.001^f^
μg/g Ct	2.72±5.50	1.39+0.89	n.s.	0.44±0.05	0.62±0.43	<0.002^d^, <0.02^f^
NI						
pre-shift							
μg/I	3.94±3.08	2.71±0.59	<0.001^e^	3.67±0.49	2.23±1.33	<0.001^d^, n.s.
μg/g Ct	2.37±2.04	2.42±1.73	n.s.	1.39±1.20	1.98±1.42	n.s., n.s.
end-shift							
μg/I	3.94±3.08	3.30±1.69	n.s.	5.34±3.06	3.30±4.06	n.s., n.s.
μg/g Ct	3.39±4.21	2.91±1.26	n.s.	3.24±3.39	3.24±3.39	n.s., n.s.
Mn						
pre-shift						
μg/I	0.69±0.49	0.49±0.18	<0.01^e^	0.34±0.08	0.40±0.17	<0.001^d^, n.s.^f^
μg/g Ct	0.51±0.51	0.47±0.29	n.s.^e^	0.18±0.06	0.36±0.20	n.s.^d^, n.s.^f^
end-shift						
μg/I	0.82±0.63	0.47±0.13	<0.001^e^	0.48±0.10	0.54±0.42	n.s.^d^, n.s.^f^
μg/g Ct	0.65±0.63	0.43±0.13	<0.01^e^	0.30±0.20	0.65±0.66	n.s.^d^, n.s.^f^
Co						
pre-shift						
μg/i	0.48±0.53	0.36±0.32	n.s.^e^	0.86±0.14	0.42±0.34	n.s.^d^, n.s.^f^
μg/g Ct	0.29±0.31	0.32±0.25	n.s.^e^	0.45±0.03	0.28±0.18	n.s.^d^, n.s.^f^
end-shift							
μg/I	0.63±0.51	0.23±0.10	<0.001^e^	0.78±0.29	0.32±0.26	<0.001^d^, n.s.^f^
μg/g Ct	0.45±0.47	0.20±0.07	<0.001^e^	0.40±0.08	0.44±0.81	n.s.^d^, n.s.^f^

Ct-creatinine.

n.s.-not significant.

Statistical significance:

a between 1 and 2;

b between 1 and 4;

c between 2 and 4;

d between 1 and 4;

e between 1 and 2;

f between 2 and 4.

An analogous trend in Cr, Ni, and Mn concentrations was observed in welders’ plasma depending on the type of respirators used. The highest concentrations of Cr, Ni, and Mn were determined in the urine of workers using a welding helmet without any respirator (M±SD 0.78±0.44 μg/l, 2.09±1.76 μg/l, and 1.77±1.17 μg/l, respectively), and statistically lowest in those using a welding helmet with powered or air fed, filtering respirator (M±SD 0.29±0.09 μg/l for Cr, p < 0.001; 1.34±0.44 μg/l for Ni, p < 0.001; 0.73±0.17 for Mn, p < 0.001) (Table 5).

### Metals relationship

Next, the authors investigated correlations between metal concentrations. The authors observed strong significant positive linear relationship correlations between Cr concentration in plasma and Cr level in urine in welders before shift expressed as μg/l as well as after Ct adjustment (r = 0.761, p < 0.0001 and r = 0.723, p < 0.0001, respectively). A similar relationship was found between plasma Cr concentration and urine Cr concentration at the end of the working shift. Linear correlation coefficients and statistical significances are provided in the Table 6.

**Table 6.** Correlation coefficient (r) between plasma and urine chromium (Cr) levels of welders in welding companies, Poland, 2018–2022

Parameter	r	p
Plasma Cr	1	0
Urine Cr		
pre-shift		
μg/I	0.761	0.0001
μg/g Ct	0.723	0.0001
end-shift		
μg/I	0.724	0.0001
μg/g Ct	0.857	0.0001

Ct – creatinine.

**Figure 1.**
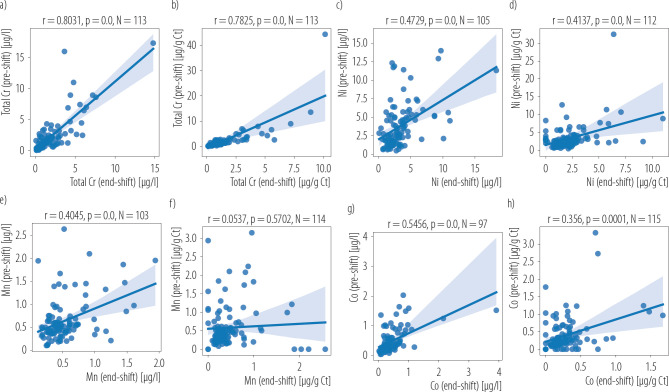
Linear correlations between a), b) chromium (Cr), c), d) nickel (Ni), e), f) manganese (Mn), and g), h) cobalt (Co) concentration in pre and end-shift urine samples of welders in welding companies, Poland, 2018–2022

There were also strong linear correlations between urinary Cr concentrations (expressed both as μg/l and μg/g Ct) before and at the end of the working shift (r = 0.803, p < 0.0001 and r = 0.782, p < 0.0001, respectively). Weaker, but statistically significant linear correlations were found between workers’ urinary Ni concentrations (μg/l) before and after the working shift (r = 0.473, p < 0.0001). Similar relationships were shown by correlating the urinary Co concentrations of welders expressed in μg/l and μg/g Ct before and after the working shift. The coefficients of linear correlation and statistical significance were as follows (r = 0.546, p < 0.0001 and r = 0.356, p < 0.0001, respectively) (Figure 1).

Ct – creatinine.

Smoking or drinking as well as length of employment had no apparent effect on the metal concentration in plasma and urine. Finally, the association between the TL and the metal concentrations was evaluated. There were no differences in mean TL values in any of the cases analyzed. The values obtained in the group of welders did not differ from those observed in the control group, and did not depend on welding techniques or the use (or not) of PPE (Table 4).

**Figure 2.**
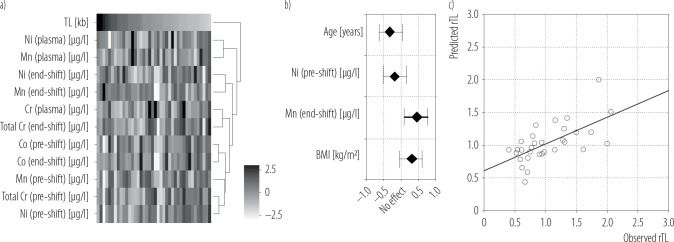
Relationship between metal concentration and telomere length: a) heatmap of Z-scores for metal concentrations relative to telomere length (TL), b) β coefficients from a multivariate linear regression model with 95% confidence intervals, c) scatter plot comparing observed and predicted telomere length with fitted linear regression line (rTL)

To this point, for each variable of metal exposition (plasma/urine), the authors first developed univariate and multivariate linear regression with clinical cofactors (age, BMI, length of employment). The diagram of analysis is provided in Figure 2.

## DISCUSSION

All welding and related processes generate a diverse mixture of fumes (airborne particles) and gases that can be hazardous to health if inhaled or ingested. The degree of hazard depends on the composition of the smoke, its concentration and the duration of exposure. The results suggest a significant impact of occupational metal exposure on welders, with increased levels of Cr, Mn, Co, and Ni observed in their biological samples (plasma and urine) compared to controls. Previous research supports these findings, showing increased amounts of aluminum, Cr, Mn, and Ni in welders. This is especially relevant as increased concentration of heavy metals is associated with various health problems like altered blood parameters, potential kidney damage, by a decrease in plasma expression of miR-21, miR-146a and miR-155, oxidative stress, inflammation, and cytotoxicity [38–40].

In the study reported in this paper, paired urine samples (both pre-shift and post-shift samples) were collected for all metals determined. The statistically increase in levels of Cr, Ni, Mn, Co in urine were demonstrated in workers suggesting recent occupational exposure to metals between the samplings (Table 2). A similar relationship was shown by Santonen et al. [41] analyzing differences between the urinary Cr concentrations of different groups of Cr-exposed workers. However, the authors’ study showed a statistically significant increase not only in Cr concentrations, as shown by Santonen et al. [41], but also in Ni, Mn, and Co concentrations indicating an accumulation of these elements in the body of welders, during a work shift.

The higher concentrations of the determined elements in the urine of workers after their working shift (Table 3) are not surprising, but may indicate an accumulation of the determined elements during the working shift, which is reflected in a higher excretion of the elements in urine. It was revealed that TIG welders had notably higher concentrations of Cr, Ni, and Mn in urine samples both before and after their shifts (Table 4). However, in the group of welders we surveyed, only 14% welded by TIG, the remainder welded by MIG (58%), and those using different welding methods during a work shift (the others) accounted for 28%. It is impossible to determine how many of the TIG welders adhered to PPE wearing regimes, which may be related to higher elemental concentrations mainly in urine (Table 4). The analysis shows that in the study group, only 27% used PPE. The results of Santonen et al. [42] showed that higher Cr exposure was observed in the group of workers exposed to Cr (bath plating), which Viegas at al. [38] links to the assumption that most of these workers did not use PPE.

The results of the study showed higher concentrations of Cr, Ni, and Mn in plasma as well as urine in welders working in confined spaces using PPE (helmet) without respirator compared to welders using protection with respirator (Table 4). This outcome is supported by studies highlighting the efficacy of PPE in decreasing exposure to welding fumes. The use of different types of respirator clearly shows that the concentrations of Cr, Ni, and Mn in both plasma and urine are significantly lower in welders using even simple protection measures such as a half mask reusable dust respirator. The data show that the highest protection against harmful welding fumes containing, *inter alia*, metal particles is provided by helmets with powered or air fed filtering respirators (Table 5).

The results of this study indicate that the use of metal helmet in welding fumes will protect the worker from some metals, but it should not be assumed that its use will provide total protection. It is accepted that air sampling to monitor metal concentrations in welding fumes/dusts should take place outside the helmet. Welders often remove their welding visors to check the weld, sampling inside the welding visor can be subject to considerable error when the welding visor is in the up position.

A study by Lehnert et al. [43] shows that metal concentrations (Cr, Ni, and Mn) measured under a welder's visor equipped with purified air supply were significantly lower than concentrations obtained outside the visor and therefore in the working environment. The authors argue that background concentrations in the area can significantly contribute to elemental concentrations in workers and point to the further need to reduce metal concentrations in the air of the working environment to better protect both welders and non-welder bystanders [43]. Moreover, the crucial role of PPE in reducing these exposures is evident, particularly helmets with aspirators, which effectively lowered plasma and urine concentrations of Cr, Ni, and Mn. This outcome is supported by studies highlighting the efficacy of PPE in decreasing exposure to welding fumes. However, the effectiveness of PPE depends on adequate training, knowledge, and safety attitudes [44,45]. Occupational exposure to xenobiotics and its effect on TL has been extensively studied in recent decades [46]. Studies have looked at occupational exposure to drugs, radiation, pesticides and many other substances [47]. Much attention was paid to exposure to metals in the environment, particularly to Pb [48], As [49] and also metals found in welding fume dusts [50]. It is now accepted that environmental exposure to xenobiotics, including occupational exposure, has an impact on TL changes [51]. It was therefore decided to investigate the relationship between exposure to metals released during the welding process and the length of TL in the blood, as a possible biomarker of effects.

The results of most of the work published to date indicate telomere shortening in populations exposed to xenobiotics. The authors’ study showed that TL expressed in relative unit in welders did not differ from that observed in the comparison group (M±SD 0.99±0.42 vs. 0.99±0.51) (Table 2). A similar range of TL values was shown in the group of welders depending on the welding method or the use of PPE (Table 4). However, it is extremely difficult to refer to the values we obtained and compare them with other authors. There are many analytical methods, mostly qPCR, but also fluorescence *in situ* hybridization (FISH) and Southern blotting. This results in the expression of TL in different, often incomparable units. Li et al. [49] showed that in women living in the high Andes (3800 m above sea level) and exposed to As, the TL expressed in relative units, ranged 0.16–0.7. In contrast Hubacek et al. [52] determined TL in workers exposed to metal nanoparticles. The TL values for exposed workers were M±SD 0.92±0.13 compared to 0.86±0.15 for controls. The values citied above are very close to the values we have obtained in the authors’ laboratory. Apparently different results were obtained by Vaiserman and Krasienkov, [53] who studied the dependence of the TL on the age of the test subject. The reported TL ranged from 4 kb to 10 kb. Similarly, Li et al. [50], who studied the TL in a group of welders, reported that M±SD values ranged from 8.7±0.84 kbp to 15.7±0.71 kbp. However, following the work of O’Callaghan [35], it can be said that the results we obtained in the study population are close to 7 kb. Thus it appears that the only option is to compare the results obtained in exposed individuals with those obtained in control group until scientific centers develop a single, uniform method for determining TL. Despite the different values of units in which TL was expressed, many studies have shown that short-term exposure to inhalable particle matters (PM_2,5_ and PM_10_) caused telomere lengthening [54]. The authors of some publications do not find a link between TL and exposure to welding fumes [50], metal nanoparticles [52], or carcinogenic wood dust [55]. Interestingly, when exposed to certain chemicals, including proven carcinogenic compounds to humans, longer telomeres were found than in the unexposed group. Examples of such compounds include As [49], benzene [56], and POPs [57].

The correlation between TL and exposure to toxic metals in human populations is unclear despite decades of observational research. Beddingfield et al. [58] reviewing 25 observational studies that considered the correlation between TL and exposure to Cd, As, Ni, selenium (Se), Pb and cesium (Cs), found that only Cd was consistently significantly correlated with shorter telomeres. The authors suggest that correlations between some metals and TL may vary across populations, and change at different levels of exposure. On the other hand in a paper published in 2024 on Cr VI exposure, the authors observed that blood Cr was negatively correlated with TL in peripheral blood cells [59].

Using an animal model to identify potential biomarkers of epigenetic changes, including TL in isolated peripheral blood mononuclear cells (PBMs) after exposure to various welding fumes, suggests that genotoxic metals (e.g., Cr and Ni) present in metal arc-stainless steel (MMA-SS) fumes, can induce markers of oxidative stress and increase TL in PBMs [60]. Some limitations of the study, e.g., the difficulty in determining a causal relationship by using a cross-sectional study design, may have affected the results obtained. Notwithstanding the limitations the final multivariate linear regression model for TL included Ni and Mn urine concentrations (pre-shift, without Ct adjustment) and clinical covariates of age and BMI (Figure 2). This model reached an r^2^ value of 0.3168 and demonstrated prediction error inversely associated with TL − the model overestimated short and underestimated longer TL.

Strikingly, the authors’ findings suggest a positive association between Ni and Mn concentrations and TL, an effect that remained statistically significant even after adjusting for age and BMI, both parameters that are somehow associated with ageing. This indicates a complex interaction between metal exposure and biological markers of ageing. May be considered that this result may be an artefact due to the very small group of participants eligible to study the effect of metal concentration on TL in the authors’ study. However, similar results were obtained in a paper by Bai et al. [61], in which the authors observe that increasing plasma Mn levels are positively associated with TL, and that multi-pollutant associations suggest that Mn is the only predictor of TL. Strengths of the authors’ study include a well-defined cohort, comprehensive assessments of metal exposure, and evaluation of different welding methods and PPE efficacy. However, limitations such as the cross-sectional design, absence of dietary metal intake data, and small study group warrant caution in interpretation. Further larger-scale longitudinal studies are necessary to better understand health impacts of heavy metal exposure and their underlying mechanisms.

## CONCLUSIONS

The authors' findings emphasize the significant metal exposure risks welders face and the crucial need for effective protective measures and ongoing monitoring to safeguard their health. The implementation of stringent safety protocols, efficient ventilation systems, and proper PPE, along with comprehensive biomonitoring programs, are essential to mitigate the adverse health effects associated with metal exposure in welding occupations [46,62].

Biological and occupational environmental monitoring can provide important, real-world information on metal concentrations in the air and in the biological material of subjects, but it does not provide information on the actual exposure of workers, especially workers using personal protective equipment. According to Santonen et al. [41], using traditional biological monitoring methods (e.g., determination of Cr in urine) can result in overestimated exposure to Cr. These values, however, may not necessarily provide much information about the actual exposure of workers if PPE is used and is effective. On the other hand, traditional biomonitoring methods (e.g., Cr in urine) may overestimate exposure because they cannot distinguish between exposure to Cr(VI) and exposure to the less hazardous chromium(III) oxide [41]. Published work to date has largely focused on studying changes in TL with exposure to a single xenobiotic. In the study of welders, it is important to bear in mind exposure to both multiple elements, UV radiation and organic compounds that may (or may not) affect TL. On the basis of the authors’ study, it cannot be claimed that TL testing can be a potential indicator of toxicity of metals welding fumes exposure.
